# Y-90 Microshperes in the Treatment of Unresectable Hepatocellular Carcinoma

**DOI:** 10.4103/1319-3767.39627

**Published:** 2008-04

**Authors:** Abdullah Al-Kalbani, Yasser Kamel

**Affiliations:** Department of Liver Transplantation, KFSH and RC, MBC-72, Riyadh 11211, Saudi Arabia

**Keywords:** Hepatocellular carcinoma, radioembolization, Y-90 microspheres

## Abstract

A small percentage of patients with hepatocellular carcinoma (HCC) are candidates for curative treatment in form of resection or transplantation. There are different treatment options for unresectable HCC-like local ablative therapies and recently systemic therapy with Sorafenib. All of these have variable response rate and had been proven to improve survival. In the last few years, there is a growing interest in TheraSphere radioembolization. It consists of yttrium90 (Y-90) embedded into nonbiodegradable glass microspheres. It is selectively administered by intraarterial hepatic injection giving high doses of radiation to the tumor and sparing the liver parenchyma. It has been shown to improve survival and used as a bridge to transplantation and to downstage tumors for resection. Therasphere seems to have favorable safety profile and has been used in patients with portal vein thrombosis with successful outcome.

Hepatocellular carcinoma (HCC) is the commonest primary malignant tumor of the liver. It is the fifth most common cancer in men and the eighth most common in women, and it ranks fourth in annual cancer mortality rates.[1]

Traditionally, patients with HCC had limited treatment options because of the locally advanced and multifocal nature of the disease. Most patients with HCC are not surgical candidates, with only 15–25% suitable for resection. Likewise, many patients with HCC do not qualify for liver transplantation because of their advanced stage of cancer at the time of diagnosis.[2]

The available treatment options for unresectable HCC like, transarterial chemoembolization (TACE), RFA, alcohol injections have variable clinical outcome and had been shown to increase survival.

Recently, Sorafenib a multikinase inhibitor was found to improve survival in patients with advanced HCC and for this reason it was approved as a treatment option in these patients.[3]

In the last few years, there is a growing interest in the use of Y-90 radioembolization in the treatment of unresectable HCC. In this short review, we will give a brief description of Y-90, patient's selection for treatment, complications of treatment, and clinical outcome.

## THERASPHERE

TheraSphere is approved by the Food and Drug Administration for treatment of unresectable HCC as well as bridge to transplantation/resection. It is approved for the treatment of liver neoplasm in other countries like Canada, India, Russia, and some European countries. It is being used as primary therapy for unresectable HCC at several large research centers in the United States.

TheraSphere consists of Y-90 embedded into nonbiodegradable glass microspheres (Mean diameter of 25 μm), in which the Y-90 is an integral constituent of the glass. Y-90 is a pure B-emitter with a physical half-life of 64.1 h. It decays to stable zirconium90. The average energy of B-emission is 0.9367 MeV, with a mean tissue penetration of 2.5 mm and a maximum of 10 mm. One gigabecquerel (27 mCi) of Y-90 per kilogram of tissue provides a dose of 50 Gy. It can be administered by intraarterial hepatic injection.

HCC tumors are generally highly vascular and receive the majority of their blood supply from the hepatic artery, compared with liver parenchyma, which receives its blood supply primarily from the portal vein. Therefore, the intraarterial injection of Y-90 microspheres represents treatment that can be delivered in a local (segmental or subsegmental), regional (lobar ***via*** left or right hepatic artery) or whole-liver (***via*** proper hepatic artery) manner, resulting in high-radiation doses to tumor while sparing liver parenchyma.[4,5]

Currently, there are two commercially available microsphere devices in which Y-90 is incorporated; one with microspheres made of glass (TheraSphere; MDS Nordion, Ottawa, ON, Canada) and the other with microspheres made of resin (SIR-Spheres; Sirtex Medical, Sydney, Australia). The SIR-Spheres use was granted approval for metastatic colorectal cancer in 2002. The two devices vary in their physical composition and radioactivity levels.

## ELIGIBILITY/CONTRAINDICATIONS

Important factors in determining eligibility for TheraSphere radioembolization include Eastern Cooperative Oncology Group performance status, liver function tests, and absence of significant lung shunting or collateral flow to the gastrointestinal tract. Patients presenting with compromised functional status with Eastern Cooperative Oncology Group scores of 2–4 are at high risk for rapid-onset of liver failure and associated morbidity with treatment. However, each patient deserves individual consideration given the favorable toxicity profile of radioembolization; some patients with poor Eastern Cooperative Oncology Group performance may still benefit from therapy.[2]

Goin ***et al***., in a retrospective study, identified the factors predictive of a 3-month mortality. Those with focal tumor, tumor volume <70%, aminotransaminases level <5 of upper limit, biluribin level <2 mg/dl, and albumin <3 g/dl, had higher 3 months mortality (49% vs. 7%).[6]

There are two absolute contraindications to microsphere-based Y-90 treatment: exaggerated hepatopulmonary shunting and reflux into the arteries that supply the gastroduodenal region.[7] This can lead to fatal and morbid complications of radiation pneumonitis and gastric ulceration.

Therefore, arteriography is essential to map the hepatic arterial supply from the celiac and the superior mesenteric artery and is the single most important test to exclude preventable complications. Using a percutaneous inserted catheter, the hepatic arteries are accessed and the supply to the liver and the adjacent gastrointestinal tract is identified. Once identified, these gastrointestinal tract arteries are coil embolized to ensure prevention of reflux of microspheres into the gut.[8]

A technetium-99 macroaggregated albumin (The 99Tc-MAA) scan is performed to estimate the dose of radiation that will be delivered to the lungs and/or viscera.[9] Patients with 99mTc-MAA evidence of potential pulmonary shunting resulting in lung doses >30 Gy should not be treated.[2]

Treatment in patients with portal vein thrombosis (PVT) had been successful.[10,11] Y-90 has recently been granted approval by the FDA for use in patients with PVT.

## COMPLICATIONS

Fatigue is the most common side effect. Fever, chills, and flu-like symptoms may also occur and generally require no specific intervention.

### Postembolization syndrome (PES):

Occurs less with Y-90 treatment than TACE. It is reported to occur three folds higher in TACE compared with TheraSpheres.[12]

Other serious complications may happen by migration of TheraSpheres to other organs causing cholycystitis, gastritis/ulcerations, pancreatitis, and pneumonitis. These complications can be avoided by proper preparation of patients as mentioned above with pretreatment mapping angiogram and the 99Tc-MAA scan.

Lymphopenia (presumed secondary to bone marrow suppression) has been noted; however, no reports of opportunistic infections complicating Y-90 therapy have been published. Finally, the development of radiation hepatitis is of concern, especially in those with decreased synthetic function identified pretherapy. This irreversible clinical entity typically presents with anicteric ascites and increased alkaline phosphatase levels. Long-term complications of hepatic fibrosis and portal hypertension have been reported.[9]

## CLINICAL OUTCOME

As a new modality, the outcome and efficacy of treatment are still in the stage of developing. No randomized controlled trials (RCTs) are published till now. The studied patient populations are inhomogeneous, different ways to assess outcome and variable end points are used; although the results seems to be promising.

The best indicator for outcome is survival, two studies showed 51–63% survival for 1 year.[7,13] This is favorable compared with the results of TACE obtained in RCTs, no trials comparing TheraSpheres to TACE have been performed.

Tumor response to treatment is noticeable in up to 100% in target lesions (in multifocal HCC) and this has a proved, indirect impact on survival but the appearance of new lesions occur in good percentage of these patients as expected.[14]

The definite advantageous field is its use in PVT which occurs in 1/3 of HCC and in hepatofugal flow when TACE is contraindicated to risk of hepatic failure induced by ischemia. The use of TheraSphere in patients with HCC and PVT offers some encouraging results.[11] As recorded median survival from date of diagnosis was 496 days in comparison with previous studies have reported that the median survival time of patients with portal venous invasion was 2.7–4 months if patient left untreated.[15]

What looks good advantage of TheraSphere is that TACE could be used after it, as there is no macroimbolization preserving the vascular access to tumor site, while radioembolization after TACE has not been successful.[16] For the microspheres to be efficacious, vascular access to the tumor capillary bed is imperative.

## CONCLUSION

The treatment of HCC still imposes a real challenge. The only proven curative treatment is surgical resection and liver transplantation. However, the majority of patients present in advanced state and therefore surgical approach is not an option in this group of patients.

Y-90 radioembolization is approved for treatment of unresectable HCC. It has been shown to have good clinical outcome and to increase survival. It is approved as definitive therapy, as bridge to transplantation and as downstaging to surgery. To date, there are no trials comparing Y-90 to TACE. However, the favorable side effects profile of Y-90 may make it a better option. Moreover, Y-90 has been shown to have a successful outcome in patients with PVT. Therefore, Y-90 has been recently approved for use in patients with PVT.
